# *Persea americana* Mill. Seed: Fractionation, Characterization, and Effects on Human Keratinocytes and Fibroblasts

**DOI:** 10.1155/2013/391247

**Published:** 2013-11-28

**Authors:** Maria del R. Ramos-Jerz, Socorro Villanueva, Gerold Jerz, Peter Winterhalter, Alexandra M. Deters

**Affiliations:** ^1^Institute of Food Chemistry, Technische Universität Braunschweig, Schleinitzstraße 20, 38106 Braunschweig, Germany; ^2^Centro de Investigación y Asistencia en Tecnología y Diseño del Estado de Jalisco (CIATEJ), 44270 Guadalajara, JAL, Mexico; ^3^Institute for Pharmaceutical Biology and Phytochemistry, University of Muenster, Hittorfstraße 56, 48149 Muenster, Germany

## Abstract

Methanolic avocado (*Persea americana* Mill., Lauraceae) seed extracts were separated by preparative HSCCC. Partition and HSCCC fractions were principally characterized by LC-ESI-MS/MS analysis. Their *in vitro* influence was investigated on proliferation, differentiation, cell viability, and gene expression on HaCaT and normal human epidermal keratinocytes (NHEK) and normal human dermal fibroblasts (NHDF). The methanol-water partition (**M**) from avocado seeds and HSCCC fraction 3 (**M.3**) were mostly composed of chlorogenic acid and its isomers. Both reduced NHDF but enhanced HaCaT keratinocytes proliferation. HSCCC fraction **M.2** composed of quinic acid among chlorogenic acid and its isomers inhibited proliferation and directly induced differentiation of keratinocytes as observed on gene and protein level. Furthermore, **M.2** increased NHDF proliferation via upregulation of growth factor receptors. Salidrosides and ABA derivatives present in HSCCC fraction **M.6** increased NHDF and keratinocyte proliferation that resulted in differentiation. The residual solvent fraction **M.7** contained among low concentrations of ABA derivatives high amounts of proanthocyanidins B1 and B2 as well as an A-type trimer and stimulated proliferation of normal cells and inhibited the proliferation of immortalized HaCaT keratinocytes.

## 1. Introduction

Originally, *Persea americana *Mill. (Lauraceae) was solely native to humid tropical areas of Mexico; it was later cultivated and extended to other regions of Latin America as well as to USA and Europe. Today, the fruits are widely grown worldwide on large scale in various subtropical countries and are generally recognized as a popular and healthy food source supplying proteins and lipids to the human diet. Three principal cultivars (Guatemalan, Mexican, and West Indian) exist with obvious differences in their fruit skin structure and size. The original Mexican wild type varieties (*span.:* aguacate criollo) are purple or black colored fruits in the size of a plum showing a smooth skin and are characterized by a yellow-green aromatic flavored flesh. The earliest record of avocado used for human nutrition was an archaeological dig in Peru that discovered avocado seeds buried with a mummy and was dated to the 8th century BC. In ethnomedicine, the avocado kernels and pulp were used to treat Saint Antonius fire (ergotism caused by fungal toxic alkaloid metabolites in grain), dander, and scabies by Mexican indigenous people [1]. An ointment made from mashed seeds had been used for women's makeup. Avocado seed oil had been applied to treat skin eruptions. Nowadays, avocado is commonly used as a fruit or as a rich source for pulp oil from fruit flesh. The oil and lipid fractions are part of various dermatological and cosmetic preparations. Furthermore, they are utilized for treatment of dry skin problems in Ayurvedic medicine [2, 3] which had been confirmed by *in vivo* studies [4, 5].

Avocado extracts, especially polyhydroxylated fatty alcohols (PFA) revealed a protection against UV-B induced damage [6] in an *in vitro* keratinocyte model. Further, AV119, a patented mixture of avocado sugars (perseitol and D-mannoheptulose [7]), increased the expression of defensins [8]. Nonsaponifiable compounds influenced collagenic structures in fibroblasts [9], and persenone A suppressed inflammation related processes [10]. Catechins, procyanidins, and hydroxycinnamic acids (from seeds and pulp) were described as antioxidative and antimicrobial agents [11]. Nevertheless, most of these components had been derived and tested from avocado fruit or leaf extracts.

The countercurrent chromatography (HSCCC) method is based on liquid-liquid partitions effect. It is successfully used for the fractionation of plants [12] as well as microorganism grown culture medium extracts [13]. A solid support is not used in the HSCCC method; therefore, sample absorption effects on stationary phase material could not occur, neither could artifact generation. More details of this technique were published by Ito [14]. This technique was used for the fractionation of avocado seeds extracts. Abscisic acid derivatives (ABA) and small polyphenols such as salidroside, as well as A-type dimers and trimers of procyanidins, were identified among others as principal natural products from a methanol extract and its methanol-water and ethyl acetate partitions from avocado seeds [15, 16]. These extracts were tested in regard to their influence on human skin keratinocytes and fibroblasts especially with respect to their ethnomedicinal background. Appropriate bioassays to investigate cellular reactions and behavior upon treatment with natural products are *in vitro* cell cultures of normal human dermal fibroblasts (NHDF), normal human epidermal keratinocytes (NHEK), and human adult low calcium high temperature keratinocytes (HaCaT keratinocytes). These cells represent the protecting dermal and epidermal layer of the human skin. These cells mainly participate in the remodeling and regeneration of skin, two crucial stages of the wound healing process as well as of skin integrity. Proliferation, differentiation, and metabolic activity of cells are important processes during skin regeneration. *In vitro* proliferation can be quantified with the 5-bromo-2′-deoxyuridine (BrdU) incorporation test. The cell viability or more precisely the metabolic activity is analyzed with diverse tetrazolium salts, which are reduced to formazan by active reductases, dehydrogenases, and reductive products of cell metabolism. The reduction of tetrazolium salts a dependency to the point of use respectively to utilized cell types [17]. Cytotoxicity was determined on basis of activity of extracellular lactate dehydrogenase (LDH). Differentiation of skin cells is obvious by morphological changes accompanied by a change in protein synthesis [18]. Therefore, the differentiation process can be measured using specific antibodies against differentiation specific proteins as involucrin [19]. To get a direction for the potential mechanism of avocado seed extract activity, gene expression analysis was carried out. Signaling elements involved in pro-proliferative, cell survival, and maturation respective differentiation were taken into consideration.

## 2. Methods

### 2.1. General

Solvents for extraction and liquid-liquid partitioning (n-hexane, methanol, and ethyl acetate) were purchased in HPLC quality from Fischer-Scientific (Schwerte, Germany). Petrolether Rotisolv (F_p_ 40–60°C) was from Carl Roth GmbH (Karlsruhe, Germany) and dichloromethane from MWG-Biotech (Ebersberg, Germany). Solvents used for the preparative HSCCC fractionation (TBME, acetonitrile, and n-butanol) were of analytical grade and were purchased from Fischer-Scientific (Schwerte, Germany), acetonitrile for LC-ESI-MS from Honeywell Speciality Chemicals (Seelze, Germany), and Nanopure water was generated by a laboratory clean water unit (Werner Reinstwasser System, diamond analytic, Leverkusen, Germany). Paper Filter no. 1 for extracts filtration was from Schleicher & Schuell (Dassel, Germany).

Standards (−)-quinic acid (**1**) were purchased from Merck-Schuchardt (Hohenbrunn, Germany) and chlorogenic acid (**16**) (3-*O*-(3, 4-dihydroxy-cinnamoyl)-*D*-quinic acid hemihydrate) from Sigma-Aldrich (Deisenhofen, Germany).

### 2.2. Extraction and Fractionation of Avocado Seeds

**Figure 1:**
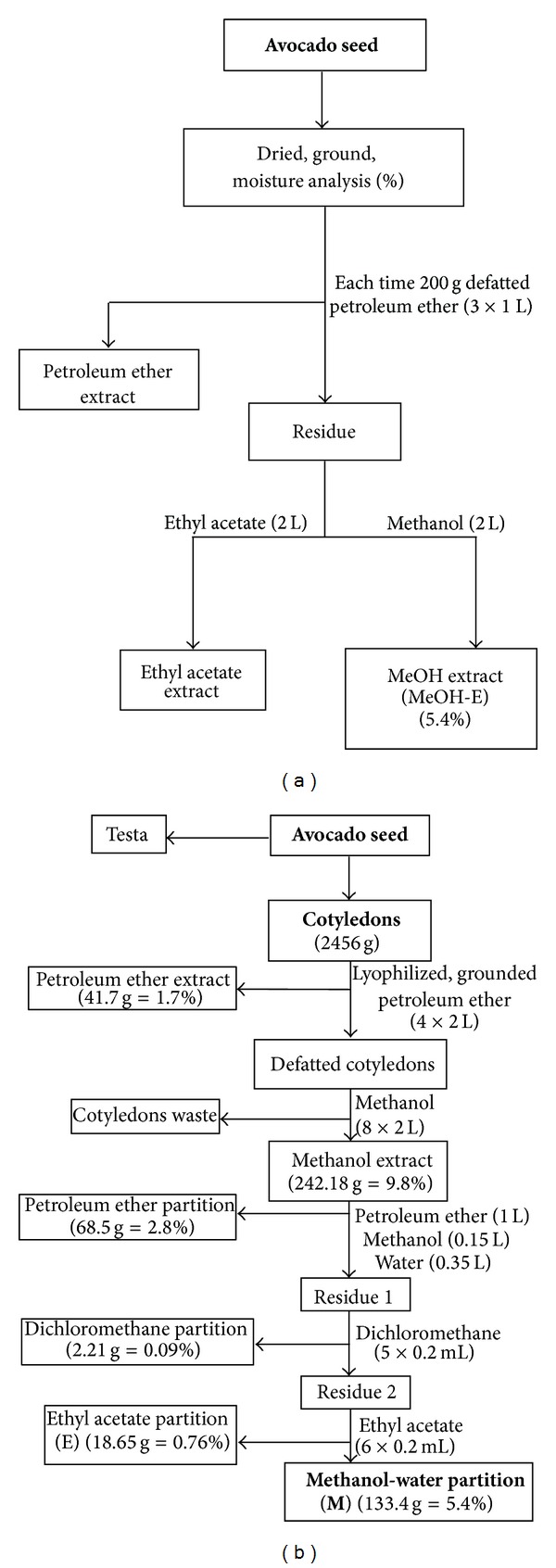
Extraction and fractionation scheme of avocado seed material. (a) Extraction of complete avocado seeds (*Persea americana* Mill. cv. *Hass*). (b) Extraction process for avocado cotyledons. Extractions as well as partitions used for HSCCC separation or *in vitro* tests are written in bold letters.

Avocado fruits (*P. americana* Mill. cv. *Hass*) were picked in a commercial orchard close to Tingüidín, Michoacán, México, in the year 2000. The seeds were released from the fruit flesh, cleaned, and entirely dried at 40°C and then vacuum packaged and stored at −20°C. For further processing the seeds were freeze-dried, powdered in a laboratory mill, and defatted with petroleum ether in multiple steps. Then the defatted avocado seeds powder was macerated exhaustively with methanol to yield the methanol extract (MeOH-E) (Figure 1(a)). In another extraction process the avocado seeds were defatted and extracted with methanol before they were extracted with distilled water and resulted in the water extract. In a second process, the seeds were separated in the cotyledons and testa. Avocado cotyledons were defatted with petroleum ether and macerated with methanol. For subsequent liquid-liquid partitioning steps, the dried methanol extract was dissolved in a mixture of Nanopure water and methanol (2 : 1, v/v) and in the first step partitioned against petroleum ether to gain the petroleum ether partition. In the second step the residual methanol-water phase was partitioned against dichloromethane to yield the semipolar compounds in the CH_2_Cl_2_ partition (not tested). In the final step ethyl acetate was used to extract polar compounds such as proanthocyanidins and other polyphenols from the methanol-water phase to yield the EtOAc partition (mrC.3r). The final residue was named MeOH-H_2_O partition **M** (Figure 1(b)). The obtained extracts and partitions were filtered through paper filters and concentrated under vacuum at 35°C. The methanol-water partition **M** was fractionated by preparative high-speed countercurrent chromatography (HSCCC) on a PTR model CCC-100 (Pharma-Tech Research Corp., Baltimore, MD, USA) equipped with three preparative coils made of a polytetrafluoroethylene tubing (2.6 mm i.d. × 165 m) connected in series. The HSCCC separation was run at a revolution speed with the biphasic solvent system consisting of *tert.*-butylmethylether-*n*-BuOH-ACN-H_2_O (1 : 3 : 1 : 5, v/v/v/v) in the *head-to-tail* mode using the lower aqueous phase as mobile phase and the upper organic phase as stationary phase. The system was operated at a spinning velocity of 1000 rpm with a flow rate of 3.0 mL min^−1^. Chromatographic UV detection of fractions was done at *λ* = 280 nm (UV trace) to give seven fractions (**M.1** to **M.7**). Compound (13) was present on **M, M.5** und **M.6**.

### 2.3. Phytochemical Analysis of Avocado Seed Compounds by LC-ESI-MS/MS

An HPLC system (pump 1100 series, autosampler 1200 series) from Agilent Technologies (B*ӧ*blingen, Germany) was coupled with an ESQUIRE LC-ESI-MS/MS ist korrekt ion-trap system from Bruker Daltonics (Bremen, Germany).

For C18 HPLC, a ProntoSil C18Aq column (5 *μ*m, 250 × 2.0 mm, Knauer, Berlin, Germany) was used. Solvent A: Nanopure water; solvent B: acetonitrile. HPLC gradient: *t* (0, 10, 40, 55, 65, 75 min), A (97, 97, 40, 0, 0, 97), and B (3, 3, 60, 100, 100, 3). Flow rate of 0.25 mL min^−1^. The HPLC separations were carried out at ambient temperature with the following conditions.

ESI-MS/MS parameter settings: drying gas was nitrogen (flow 9.0 L min^−1^, 310°C), and nebulizer pressure was set to 40 psi. ESI-MS/MS ionization parameters (negative mode): capillary 3500 V, end plate off set 500 V, capillary exit −94.6 V, trap drive 35, ICC target 50000, maximum accumulation time 200 ms, charge control on, scan range *m/z *50–2200, threshold auto MS/MS 500, MS/MS experiments afforded a fragmentation amplitude value of 1.2 V.

ESI-MS (negative ionization mode) and MS/MS fragmentation data with [M-H]^−^ signals of the identified compounds in the crude extract, solvent partitions, and HSCCC fractions: (−)-Quinic acid (**1**): ESI-MS: *m/z* 191, MS/MS: *m/z *173, 129, 111, 100, 85. Hydroxy-salidroside (**13**): ESI-MS: *m/z *315, MS/MS: *m/z *179, 161, 153, 135, 119, 113, 89. Chlorogenic acid [syn. 3-*O*-(3, 4-dihydroxycinnamoyl)-*D*-quinic acid] (**16**): ESI-MS: *m/z* 353, MS/MS: *m/z *191, 179, 135. Tyrosol-1′-*β*-D-*O*-glucoside (salidroside) (**17**): ESI-MS: *m/z* 299, MS/MS: *m/z *179, 161, 143, 131, 119, 113, 101, 89.  (1′*R*, 3′*R*, 5′*R*, 8′*S*)*-epi-*dihydrophaseic acid  *β*-D-glucoside (**18**): ESI-MS: *m/z* 443, MS/MS: *m/z* 425, 237, 281, 161. Proanthocyanidin dimer B1 (**19**) and B2 (**20**): ESI-MS: *m/z *577, MS/MS: *m/z *407, 425, 289. (1′*S*,  6′*R*)-8′-hydroxyabscisic acid  *β*-D-glucoside (**21**): ESI-MS: *m/z* 441, MS/MS: *m/z* 397, 330, 139, 161. The trimers proanthocyanidin A2-(+)-catechin or proanthocyanidin A2-(−)-epicatechin (**24**) occurred as trace: ESI-MS: *m/z *863, MS/MS: *m/z *289. ESI-MS and fragmentations patterns (MS/MS) (*m/z*) of not identified compounds are described in Table 3.

### 2.4. Skin Cell Culture

All chemicals used for bioassays were of analytical quality and were purchased from Diagonal (Muenster, Germany). Cell culture media supplements were from PAA (Coelbe, Germany) as not mentioned otherwise.

Confined cell lines were obtained after isolation of fibroblasts (NHDF) and keratinocytes (NHEK) from human skin, obtained from skin surgery (University Hospital of Muenster, Germany, Department of Dermatology, Department of Pediatrics) of various Caucasian subjects. The study was approved by the local ethical committee of University of Muenster (acceptance no. 2006-117-f-S). NHDF and NHEK were isolated and cultivated as described earlier [20]. The continuous keratinocytes cell line of immortalized HaCaT keratinocytes, kindly provided by Prof. Fusenig (German Cancer Research Institute, Heidelberg, Germany), were cultivated with D-MEM high glucose (10% FCS, 1% penicillin/streptomycin, 1% glutamine, and 1% nonessential amino acids) in an 8% CO_2_ humidified atmosphere. HaCaTs were used because there were not enough NHEK at that time to do every experiment in three replicates with normal keratinocytes. For investigations during the 2nd to 6th passage (confined cell lines) and 56th–60th passage (HaCaT) the cells were directly adapted to serum-free media (test medium); fibroblasts to MEM high glucose, SerEx (10%), and L-glutamine (1%); and keratinocytes (NHEK and HaCaT) to MCDB153 complete. For gene expression studies cells were shortly adapted to basal media supplemented only with L-glutamine but without growth factors, BPE, and serum (minimal medium).

### 2.5. Influence of Test Compounds on Skin Cell Response

All tests were performed in 96-well plates (Sarstedt, Nuembrecht, Germany) at starting cell densities of 5 × 10^3^ keratinocytes and 3 × 10^3^ fibroblasts/well. Extracts, cotyledon solvent partitions, and HSCCC fractions of *P. americana* were solved into a stock solution of 1 mg/mL in Aqua Millipore, sterilized by filtration through a 0.2 *μ*m regenerated cellulose acetate membrane, and solved in the recommended serum-free media to a final concentration of 10 *μ*g/mL. As positive control 10% FCS was added to test medium [21]. Incubation started 24 h after seeding when the cells had reached a confluence of 50% and ended 48 h later by adding 5-bromo-2′-deoxyuridine (BrdU), 3-(4, 5-Dimethyl-2-thiazolyl)-2, 5-diphenyl-2H-tetrazolium bromide (MTT), and (4-[3-(4-Iodophenyl)-2-(4-nitrophenyl)-2H-5-tetrazolio]-1, 3-benzene disulfonate (WST-1). BrdU incorporation assay, WST-1 and LDH assays were performed according to the manufacturer's instructions (Roche, Penzberg, Germany). Activity of intracellular reducing enzymes was measured by MTT test. Terminal differentiation of NHEK was elucidated as described earlier [20].

### 2.6. Gene Expression Analysis of Keratinocytes and Fibroblasts

Gene expression of genes related to proliferation and differentiation was investigated after incubation of skin cells with *P. americana* seeds extracts for 6 and 24 h in basal media. Appropriate for investigation of, respectively, differentiation and maturation related cell signaling were SMAD3, a member of a protein family that are homologs of both the Drosophila protein, mothers against decapentaplegic (MAD), and the *Caenorhabditis elegans* protein SMA, which participate in TGF-*β* signaling [24] and involucrin [25], was appropriate for investigation of differentiation respectively maturation related cell signaling. Procollagen 1*α*2 (Col1A2; [26]) and fibronectin 1 [27], both being proteins of extracellular matrix (ECM) as well as the epidermal growth factor receptor (EGFR [28]), were chosen because of their partition on wound healing respective proliferation related processes. 18s RNA was used as endogenous control since GAPDH regulated itself during the keratinocyte differentiation process [29]. Total RNA was isolated by InnuPREP RNA Mini Kit (Analytik Jena, Jena, Germany). After qualitative and quantitative analysis of RNA at 260 nm RNA was transcribed to cDNA by High-Capacity cDNA Reverse Transcription Kit (Life Technologies, Darmstadt). cDNA was diluted with RNa free water to 20 ng cDNA. RT-PCR was performed by TaqMan gene expression assays (20×; Life Technologies, Darmstadt, Germany) described in Table 1. Experiments were performed with TaqMan Universal MasterMix 2× without AmpErase (Life Technologies, Darmstadt, Germany) on a 7300 Real-Time PCR system (Life Technologies, Darmstadt, Germany). Gene expression was calculated with the comparative Ct method. In step one a normalization of target gene occurred to the endogenous control 18s RNA (ΔCt), followed by normalization of the normalized sample ΔCt to the normalized calibrator sample ΔCt (untreated NHEK) in step two (ΔΔCt). The relative values were obtained using the formula  2^−ΔΔCt^.

### 2.7. Statistical Analysis

Statistical evaluation was performed by Dunnett's post hoc test after ANOVA for comparison of three to four treatment groups after variance calculation by Levene. The results were considered significant with  *P*  values less than 0.05.

## 3. Results

### 3.1. Phytochemical Analysis of Avocado Cotyledons

**Figure 2:**
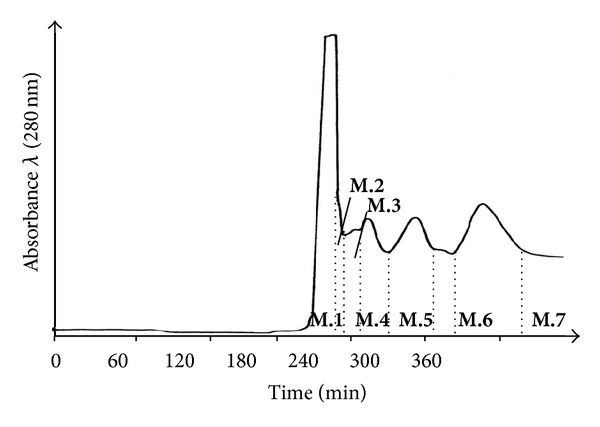
HSCCC-separation of the methanol-water partition **M** from avocado seed cotyledons material. Recovered fractions **M.1** to **M.6** and the coil fraction **M.7** by using the polar biphasic solvent system: TBME-*n*-BuOH-ACN-H_2_O (1 : 3 : 1 : 5, v/v/v/v) (three runs between 1-2 g injection amount were made).

The methanol-water partition **M** yielded 5.4% of dried avocado cotyledons. 5.4 g of methanol-water partition **M** were further separated by several reproducible preparative high-speed countercurrent chromatography (HSCCC) and resulted in six fractions (**M.1** to **M.6**) and the additional residual solvent fraction coil fraction **M.7** containing the more apolar components which were not eluted from the HSCCC column coil system (Figure 2). Fraction number 2 (**M.2**) was obtained with the highest yield followed by **M.7**, **M.6,** and **M.3** (Table 2).

**Figure 3:**
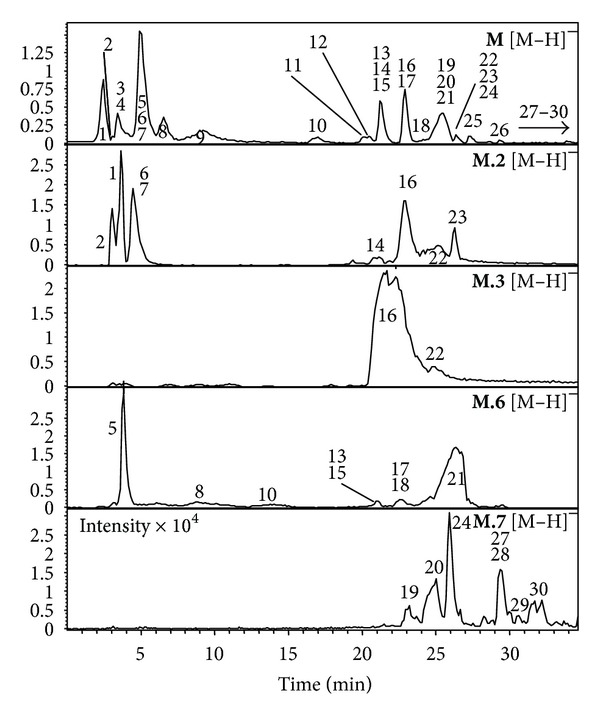
LC-ESI-MS (negative mode) of the methanol-water partition **M** and the recovered HSCCC fractions (**M.2**, **M.3**, **M.6,** and **M.7**). Intensity × 10^5^ when not another is written.

Methanol-water partition **M** as well as the HSCCC fractions had been shown to be active in the *in vitro* tests. They were analyzed by negative LC-ESI–MS (Figure 3). Secondary plant metabolites from avocado seeds in the mass range *m/z* 191 to *m/z* 863 were detected (Table 3).

The occurrence of abscisic acid derivatives such as (1′*S*,  6′*R*)-8′-hydroxyabscisic acid  *β*-D-glucoside (**21**) and *( *1′*R, *  3′*R, *  5′*R, *  8′*S)-epi-*dihydrophaseic acid  *β*-D-glucoside (**18**) was confirmed by LC-ESI-MS analysis with [M-H]^−^ signals at *m/z* 441 and *m/z* 443 and indicated by their characteristic MS/MS fragmentation data (397, 330, 161 and 425, 237). The structural identity of these compounds was elucidated before in avocado seed extracts by 1D/2D-NMR experiments [15]. These compounds were then identified by retention time and MS/MS data in the HSCCC fraction **M.6** which was used for the bioassays. The low molecular weight polyphenol tyrosol-1′-*β*-D-O-glucoside (salidroside) (**17**) was identified in the methanol soluble avocado seed extract and in the cotyledons extract. The constitution of **17** and hydroxy-salidroside (**13**) was also isolated and elucidated before by preparative HSCCC isolation from other fractions of avocado seeds [16].

**Figure 4:**
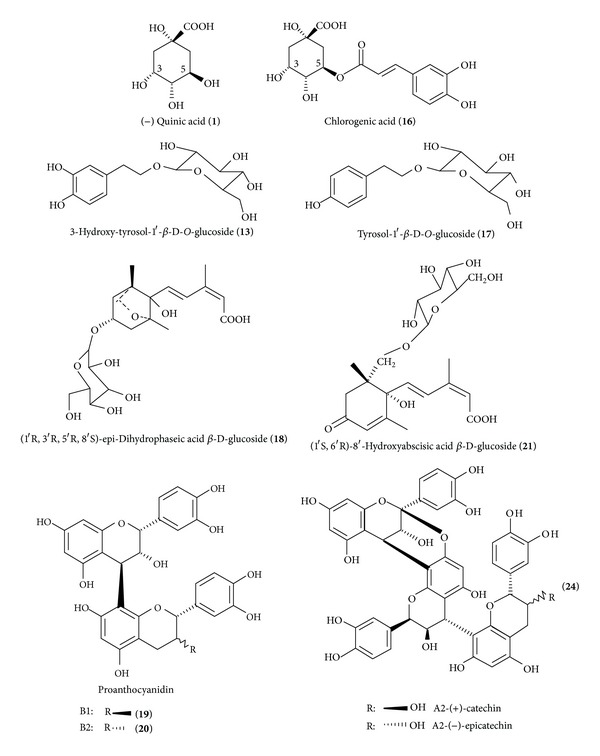
Structures of the identified compounds by LC-ESI-MS analysis in the avocado seeds extracts and the tested HSCCC fractions.

The proanthocyanidin B1 (**19**) and B2 dimers (**20**) and A-type linked trimer (**24**) were identified in the fractions **M.7** by selected [M-H]^−^ ion traces at *m/z* 577 and *m/z *863, respectively, and were in accordance with published data [16] (Figure 4).

With standards of (−)-quinic acid (**1**) (with [M-H]^−^ at *m/z* 191) and chlorogenic acid (3-*O*-(3,4-dihydroxycinnamoyl)-*D*-quinic acid; **16**) with [M-H]^−^ at *m/z* 353 it was confirmed that these compounds were present in the HSCCC fractions **M.2** and **M.3**. Additional peaks at [M-H]^−^ at *m/z* 353 were detected and indicated positional isomers of **16**, such as 4- and 5-caffeoylquinic acid which were not distinguished by MS/MS fragmentation data.

### 3.2. Influence on Fibroblasts

**Figure 5:**
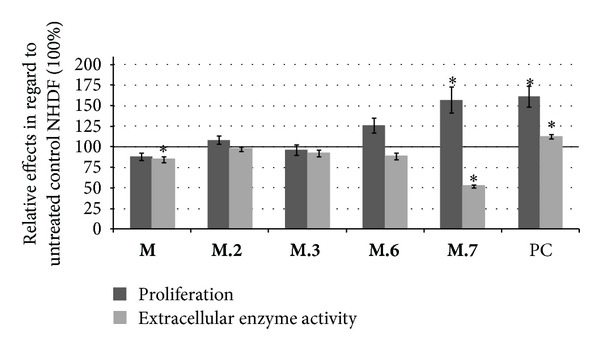
Effects of 10 *μ*g/mL avocado cotyledon methanol-water partition and HSCCC fractions on the fibroblasts proliferation (dark columns) determined by BrdU incorporation assay and extracellular enzyme activity calculated with WST-1 reduction assay (bright columns). Values were normalized to proliferation and enzyme activity of untreated NHDF (= 100%, —). (Error bars = SE;  *n* = 24;  *P < 0.05  to untreated NHDF; PC: positive control = 10% FCS).

NHDF differently responded to the incubation with the avocado cotyledon methanol-water partition **M** and HSCCC fractions. The HSCCC fractions **M.2**, **M.6,** and **M.7** enhanced the proliferation rate of NHDF compared to untreated cells. A significant increase of proliferation rates compared to untreated NHDF was observed after treatment with **M.7**. HSCCC fraction **M.6** enhanced the average proliferation rate considerably but not significantly, and **M.2** only showed a marginal effect. An insignificant reduction of proliferation was seen when NHDF were incubated with the methanol-water partition **M** and HSCCC fraction **M.3** (Figure 5). Metabolic activity was analyzed by reduction of WST-1, a formazan that is not able to penetrate the cell membrane. WST-1 reduction through extracellular active reductive enzymes was reduced after treatment with the methanol-water partition **M** and each HSCCC fraction. In case of **M** and **M.7** there was a significant inhibition of extracellular enzyme activity (Figure 5).

Cytotoxic effects were investigated on the base of LDH activity in culture supernatants as a result of cell membrane damage. The LDH activity of cells incubated with avocado cotyledon fractions was normalized to the naturally occurring LDH release of untreated cells. As shown in Table 4 no cytotoxic effects were observed when fibroblasts were incubated with 10 *μ*g/mL of avocado cotyledon methanol-water partition **M** and HSCCC fractions.

Gene expression analysis revealed that the methanol-water partition **M** and fraction **M.3** induced slightly the gene expression of fibronectin (FN1) and collagen 1*α*2 (Col1A2), respectively, but none of the other genes whose expression was analyzed after 6 h of incubation. Fraction **M.2** increased the gene expression of epidermal growth factor receptor (EGFR), insulin receptor (InsR), signal transducer and activator 6 (STAT6), and Col1A2 and FN1. Treatment of NHDF with HSCCC fraction **M.6** induced the gene expression of FN1 but inhibited gene expression of SMAD3. HSCCC coil fraction **M.7** treated NHDF revealed an upregulated gene expression of InsR and FN1. A slight stimulation of EGFR, STAT6, SMAD3, and Col1A2 gene expression was also observed, but it was not possible to do a statistically firm calculation of this effect because of issues with adequate endogenous control (Table 5).

### 3.3. Influence on Keratinocytes

**Figure 6:**
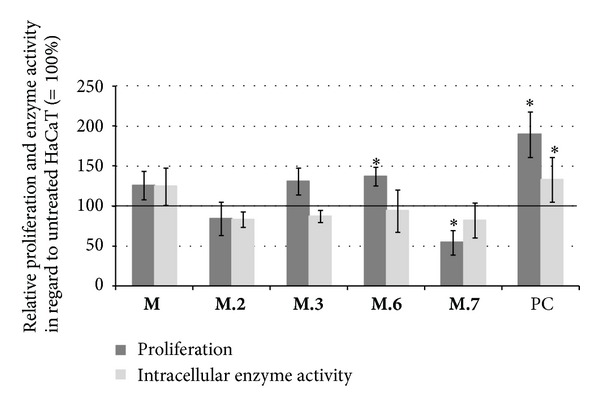
Effects of 10 *μ*g/mL avocado cotyledon methanol-water partition **M** and HSCCC fractions on HaCaT keratinocytes. Proliferation rates (dark columns) were investigated with the BrdU incorporation assay; the intracellular metabolism (bright columns) was measured through MTT reduction. Values were normalized to proliferation and accordingly intracellular metabolism of untreated HaCaT (=100%, —). (Error bars = SE;  *n* = 24;  *P < 0.05  to untreated NHDF; PC: positive control = 10% FCS).

The methanol-water partition of avocado cotyledons **M** slightly increased the proliferation rate and intracellular enzyme activity of HaCaT keratinocytes. The HSCCC fractions **M.3** and **M.6** enhanced the proliferation. In case of **M.6** the promotion of proliferation was significant. While HSCCC fraction **M.2** insignificantly inhibited the HaCaT proliferation, a significant inhibition was observed when HaCaTs were treated with **M.7** (Figure 6).

The cell metabolism was not affected by the methanol-water partition **M** but each of tested HSCCC fractions slightly inhibited the reduction of MTT, whereas **M.6** had the least influence (Figure 6). Methanol-water partition **M** and HSCCC fraction **M.3** did not influence the LDH activity in culture supernatants. Incubation of HaCaT with fractions **M.2**, **M.6,** and **M.7** increased the extracellular LDH activity in a range between 18% (**M.7**) and 25% (**M.2**; Table 4).

**Figure 7:**
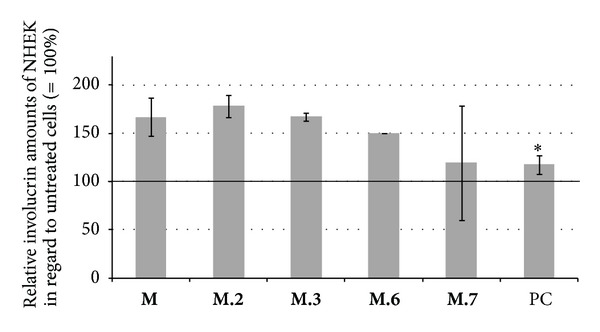
Involucrin amounts of NHEK incubated with 10 *μ*g/mL avocado cotyledon methanol-water partition **M** and corresponding HSCCC fractions for 9 days. Values were normalized to involucrin amounts of untreated NEHK (=100%, —). (Error bars = SE;  *n* = 12;  *P < 0.05  to untreated NHEK; PC: positive control = 13 *μ*g/mL A23187 in 2 mM calcium containing medium).

Normal human epidermal keratinocytes (NHEK) were incubated with 10 *μ*g/mL avocado cotyledon methanol-water partition **M** and HSCCC fractions for nine days to investigate the keratinocyte differentiation. NHEK were chosen since the used HaCaTs did not differentiate in several tests. After extraction of total proteins the amount of the differentiation specific protein involucrin was semiquantitatively analyzed using Dot blot technique. As shown in Figure 7 the methanol-water partition **M** and the corresponding HSCCC fractions **M.2**, **M.3,** and **M.6** increased the synthesis of involucrin. Incubation of NHEK with the HSCCC “coil fraction” **M.7** in three independent tests resulted in high standard deviations and differences between results. When data of each test were pooled it got obvious that the average involucrin expression was marginal elevated compared to pooled data of untreated cells (Figure 7).

For preliminary gene expression analysis NHEK were used instead of HaCaT because NHEK give an account of the situation in normal skin. First results revealed minor changes in gene expression compared to the untreated cells after 6 h. Prolonging the incubation time for 18 h mostly resulted in a stimulation of gene expression. Methanol-water partition **M** exhibited the least influence but the expression of KGFR (FGFR2b) was enhanced. To minor extend genes of EGFR and STAT6 were upregulated. HSCCC fraction **M.2** treated cells showed an increased gene expression of KGFR (FGFR2b), EGFR, and SMAD3. No statistically firm results were obtained with **M.3** and **M.7** because the 18s RNA was also influenced and a normalization was not possible. **M.6** mostly increased the gene expression of STAT6 and insignificantly enhanced the expression of EGFR (data not shown).

## 4. Discussion

For the preparative fractionation of the methanol-water partition **M** from avocado cotyledons, high-speed countercurrent chromatography (HSCCC) resulted in high quantities of the fractions later used for the biological assays, further isolation, and spectroscopical elucidation of the active compounds.

Chlorogenic acid (3-CQA **16**) and maybe its 4-, 5-CQA isomers were present in the methanol-water partition **M** and in recovered HSCCC fractions **M.2** and **M.3**. Hydroxy-salidroside (**13**) and tyrosol-glucoside (*syn.*: salidroside **17**) were present in M and HSCCC fraction **M.6** as well as derivatives of abscisic acid glucosides (ABA, **18**, **21**). HSCCC “coil fraction” **M.7** was mostly composed of dimers and trimers of proanthocyanidins, but also ABA derivatives **18** and **21** were regained in traces.

Biological assays revealed a relation between composition and effects of methanol-water partition **M** and its corresponding HSCCC fractions and a dependency on the used cell type. Methanol-water partition **M** slightly reduced the proliferation rates of NHDF but slightly stimulated the proliferation of HaCaT, an effect that was also observed with NHEK in one preliminary experiment (data not shown). The enhanced proliferation resulted in a contact inhibition that explains the differentiation of NHEK after 9 days of incubation [18]. The results obtained with functional *in vitro* tests went conform with gene expression results. Every investigated gene that is related to proliferation in NHDF was not affected, while in NHEK the KGFR gene, responsible for stimulation of keratinocytes' proliferation, was upregulated [30]. HSCCC fraction **M.2** composed of quinic acid (**1**) and chlorogenic acid (**16**) exerted a slight proliferation stimulating activity on NHDF but did not affect metabolic activity and cytotoxicity of fibroblasts. Gene expression analysis revealed an upregulation of genes that are involved in proliferation (STAT6 and EGFR), cell survival (InsR), and/or maturation processes (SMAD3, Col1A2, and FN1). This leads to the assumption that the two main components quinic acid and chlorogenic acid exert different activity on fibroblasts. Quinic acid might be responsible for the pro-proliferative effect because chlorogenic acid was shown to inhibit growth of fibroblasts [31] and because **M.3**, which did not contain quinic acid, inhibited the NHDF proliferation. Furthermore, it was previously described that quinic acid inhibited NF_k_B signaling and stimulated DNA repair mechanism [32]. On the other hand, **M.2** slightly inhibited the proliferation and the metabolic activity of HaCaT and directly induced the differentiation of NHEK. The upregulation of SMAD3 went conform with the direct induction of differentiation. SMAD3 is part of signal pathways that are related to cellular development respective differentiation [24]. In addition to their effect on cellular proliferation EGFR and KGFR (FGFR2b) are involved in the regulation of keratinocyte differentiation [30, 33]. In this context the coexpression of EGFR and KGFR (FGFR2b) also supports the obtained results of functional tests. Quinic acid and respective or chlorogenic acid differently affected HaCaT keratinocytes compared to fibroblasts. This outcome was more considerable when results obtained with HSCCC fraction **M.3** were regarded. Due to the absence of further information it was difficult to allocate the effect to quinic acid or chlorogenic acid. A circumspective suggestion is that quinic acid inhibited the HaCaT proliferation because the absence of quinic acid in **M.3** results in a clear increase in HaCaT growth. Additionally, chlorogenic acid, the main component of **M.3**, was already reported to stimulate the proliferation of other epithelial cell cultures [34].

As detected by LC-ESI-MS the HSCCC fraction **M.6** was principally composed of salidroside (**17**), hydroxy salidroside (**13**), ABA derivatives (**18**, **21**), and structurally unknown compounds. **M.6** was the most active fraction in the functional assays because it significantly increased the proliferation rates of both keratinocytes and fibroblasts. Gene expression analysis showed that **M.6** promoted the proliferation of NHDF and NHEK via different signal pathways. In NHDF the repression of SMAD3 might indicate an inhibition of maturation related signal pathways and a preferred induction of signal transduction resulting in proliferation. The increased expression of fibronectin 1 gene led assume that **M.6** influenced NHDF proliferation via the ECM and not directly via growth factor receptors since neither the EGFR, InsR, nor STAT6 were affected. Therefore, the induction of proliferation took place through remodeling of ECM proteins [35].

In NHEK an induction of EGFR and STAT6 gene expression was observed after 24 h. As mentioned before, EGFR is involved in proliferation and differentiation processes [30, 33]. From this point of view the slight increase in EGFR gene expression is jointly responsible for the increase of HaCaT and NHEK proliferation rates. The STAT6 pathway is usually affected by IL-4 and IL-13, but there are reports that the activation occurred upon a different stimulus [36]. Nevertheless, the pro-proliferative effect of **M.6** is based on the inhibition of pathways that prevent proliferation. The effect of **M.6** on skin cells might base on the ABA derivatives **18** and **21**. As shown in Figure 3,  **21** represents the component with the highest amount, and additionally both **18** and **21** resemble autocrine abscisic acid that acts as stress response hormone in animal cells [37]. In contrast it has been shown that ABA derivatives had no influence on healthy fibroblasts [38] and that ABA induction of cellular response is based on the activation of ADP-ribose and an increase in intracellular  Ca^2+^  concentrations [39]. The latter would result in an induction of keratinocyte differentiation, but this was not observed during the tests. Among ABA derivatives salidroside (**17**) and hydroxy-salidroside (**13**) were detected in **M.6**. Salidroside is reported to inhibit the proliferation of cancer cells [40], but there are as much reports that show an antiapoptotic effect [41] and pro-proliferative effect especially on fibroblasts [42, 43]. Furthermore, salidroside attenuates the level of intracellular calcium [44], resulting in a support of keratinocyte proliferation. Supernatants might relate to the increased cell number particularly with regard to the unaltered reduction of MTT and WST-1 test compared to untreated cells.

HSCCC fractionation “coil fraction” **M.7** was mostly composed of proanthocyanidin B1 and B2 as well as an A-type linked trimer among ABA derivatives and not yet identified components. Results showed that the difference in composition was recovered in bioactivity of **M.7** on human skin cells. Such an apparent difference in susceptibility to natural products of keratinocytes and fibroblasts has been shown with the other fractions but not in such an apparent way. **M.7** steeply increased NHDF proliferation, did not affect proliferation and intracellular enzyme activity of normal keratinocytes (data not shown) but significantly inhibited HaCaT proliferation, increased LDH activity in supernatant, and slightly reduced the intracellular enzyme activity of HaCaT. Unfortunately the analysis of NHEK gene expression failed, and no gene expression analysis was done with HaCaT because they exert a modified gene expression compared to NHEK. Nevertheless, the increase of NHDF proliferation was accompanied with an increase of insulin receptor and FN1 gene expression. These results indicate that **M.7** influences the cellular survival mechanisms of fibroblasts and the signal transduction via ECM. The question for the responsible ingredient of **M.7** is not clarified yet. As with fractions **M.2** and **M.6** there are two different classes of bioactive ingredients and not identified components. On the one hand, there are traces of ABA derivatives which were also present in **M.6**. On the other hand, **M.7** predominantly contained the proanthocyanidins B1 and B2 as well as A-type linked trimers. Results obtained with **M.7** are not comparable with bioactivities previously described of ABA derivatives. But HSCCC “coil fraction” **M.7** acted as being selective on normal cells (NHDF and NHEK) and on immortalized HaCaT cells as a synthesized procyanidin, which was earlier reported Kim et al [45]. Furthermore, the inhibitory activity on fast growing cells like HaCaT was shown for proanthocyanidin trimers [46]. Further previous studies on the bioactivity of proanthocyanidins support the recent findings. Procyanidin B2 was found to induce the proliferation of different normal cells [47, 48], and redox-active proanthocyanidins of grape seeds improve the dermal wound healing [49]. Additionally, procyanidolic oligomers interacted with the ECM proteins of fibroblasts [50], and such an interaction might base the upregulation of fibronectin gene expression that was measured after treatment of NHDF with **M.7**.

In conclusion the recent results show that the investigated avocado extracts offered a high impact on cellular function like proliferation, enzyme activity, and differentiation of skin cell that base on an influence on their gene expression. The effects of HSCCC fraction differed due to their composition and the cell type they were tested on. Although further investigation must prove the following assumption, the bioactivity of avocado seed extracts was related to quinic acid, chlorogenic acid, salidrosides, ABA derivatives, and proanthocyanidins. In view of traditional use and antiproliferative activity of **M.7** especially on immortalized HaCaT cells the results present a sound baseline for a successful development of new medications. Also the activities of HSCCC fractions **M.2** and **M.6** are worthy to be investigated in regard to their properties to improve wound healing.

## Figures and Tables

**Table 1:** Assay IDs (Life Technologies, Darmstadt) for gene expression analysis performed with Real-Time PCR. NHDF and NHEK were treated with 10 *µ*g/mL avocado seed HSCCC fractions. 18 s rRNA was used for endogenous control. KGFR and involucrin were only used for analysis of NHEK gene expression, while fibronectin 1 and collagen 1 gene expression was only measured in NHDF.

Investigated genes belong to	Abbreviation	NHDF	NHEK
Proliferation	STAT6	Hs00598618m1	
	InsR	Hs00169631m1	
	KGFR (FGFR2b)		Hs00240796m1

Proliferation or differentiation (depending on stimulus)	EGFR	Hs01076068m1	
	FN1	Hs00415006_m1	

Differentiation	Col1A2	Hs00164099_m1	
	Inv		Hs00846307s1
	SMAD3	Hs00706299s1	

Endogenous control	18 s	Hs99999901s1	

**Table 2:** Yields of whole avocado seed extracts, cotyledon solvent partitions, and HSCCC fractions. Specific compounds were identified by LC-ESI-MS/MS and 1D/2D-NMR spectroscopy [16].

Avocado seed fractions	Amounts [mg/%]	Identified compounds/composition
Methanol-water partition **M**	[133 g/5.4%*]	1, **13**, 16, **17, 18**, 19, 20, **21**, 24, and ni
**M.2**	[1440 mg/1.4%*]	1, 16 and its isomers; ni
**M.3**	[100 mg /0.10%*]	16 and its isomer; ni
**M.6**	[87 mg/0.22 %*]	13, 17, 18, 21, and ni
**M.7**	[743 mg/0.74%*]	19, 20, and 24; traces of **18 **and **21**; and ni

*yield from dried cotyledons; ni: not identified.

**Table 3:** Not identified metabolite profile in the methanol-water partition **M** and in the respective HSCCC fractions.

Signal	Molecular ion *m*/*z* [M–H]^−^ fragmentation pattern (MS^2^)
2	211
3	342 (179, 161, 119, 101)
4	443 (219, 161)
5	353 (191, 179, 135)
6	191
7	383
8	337 (191, 163, 119)
9	353 (191, 179, 135)
10	353 (191, 179, 135)
11	397 (251, 163, 125)
12	397 (251, 163)
14	433
15	353 (315, 191, 173, 135)
22	353 (191, 179, 135)
23	387 (369, 207, 179, 163, 147)
25	415 (149, 358, 311)
26	409 (263, 161)
27	577, 427
28	445 (410, 267)
29	863
30	497 (461, 383)

**Table 4:** Percentage cytotoxicity of avocado cotyledon products determined by LDH activity in cell culture supernatants normalized to the LDH release of untreated cells (=100%).

Avocado seed products	NHDF [%]	HaCaT [%]
Methanol-water partition **M**	103	103
**M.2**	101	125
**M.3**	102	100
**M.6**	102	120
**M.7**	99	118

**Table 5:** Regulation of NHDF gene expression after incubation for 6 h with 10 *µ*g/mL avocado cotyledon methanol-water partition **M** and HSCCC fractions in minimal media. Gene expression of three independent tests was normalized to the endogenous control and to the target gene expression of untreated cells. A > 2-fold up- or downregulation has been considered as significant. Significant values are highlighted in bold letters; n.c.: a statistically firm calculation was not possible.

	EGFR	InsR	STAT6	SMAD3	Col1A2	FN1
Methanol-water partition **M**	1	1	1	1	1	2
**M.2**	**8**	**7**	**4**	**4**	**10**	**5**
**M.3**	1	1	1	1	2	1
**M.6**	1	2	1	0.5	2	**3**
**M.7**	n.c.	**3**	n.c.	n.c.	n.c.	**6**

## Glossary

FCS: Fetal calf serum
HaCaT: Human adult low calcium high temperature keratinocyte
NHDF: Normal human dermal fibroblasts
NHEK: Normal human epidermal keratinocytes
EGFR: Epidermal growth factor receptor
InsR: Insulin receptor
KGFR (FGFR2): Keratinocyte growth factor receptor or fibroblast growth factor receptor 2
STAT6: Signal transducer and activator of transcription 6
SMAD3: Drosophila protein, mothers against decapentaplegic (MAD) and the *Caenorhabditis elegans* protein (SMA), homolog 3
FN1: Fibronectin 1
Col1A2: Procollagen 1*α*2
ECM: Extracellular matrix
LDH: Lactate dehydrogenase
BrdU: 5-Bromo-2′-deoxyuridine
MTT: 3-(4, 5-Dimethyl-2-thiazolyl)-2, 5-diphenyl-2H-tetrazolium bromid
WST-1: Water-soluble tetrazolium (4-[3-(4-iodophenyl)-2-(4-nitrophenyl)-2H-5-tetrazolio]-1, 3-benzene disulfonate.
